# Effect of a Brief Structured Intervention on Perceived Stress and Coping Patterns Among MBBS Students During Clinical Posting: A Pre-Post Interventional Study

**DOI:** 10.7759/cureus.115205

**Published:** 2026-08-26

**Authors:** Paulraj Rajavel Murugan, Thenrajan Padmavathi, Abiselvi Annasekaran

**Affiliations:** 1 Department of General Medicine, Government Thoothukudi Medical College, Thoothukudi, IND; 2 Institute of Health Profession Education, Sri Balaji Vidyapeeth, Puducherry, IND; 3 Department of Pharmacology, Kanyakumari Government Medical College, Kanyakumari, IND; 4 Department of Community Medicine, Government Thoothukudi Medical College, Thoothukudi, IND

**Keywords:** brief cope, clinical posting, coping strategies, medical students, perceived stress, pre-post intervention

## Abstract

Background

Clinical postings expose undergraduate medical students to considerable academic and emotional stress, which may adversely affect learning, wellbeing, and professional development. Brief structured interventions incorporating coping skills training have been proposed to mitigate this stress, but data from resource-constrained clinical training settings remain limited. This study aimed to evaluate the effect of a brief structured intervention on perceived stress and active coping among undergraduate MBBS students during their Medicine clinical posting.

Methodology

A pre-post interventional study was conducted among 50 undergraduate MBBS students undergoing Medicine posting at a government medical college. Participants underwent a brief structured program comprising sessions on understanding clinical stress, problem-solving and time-management skills, and basic emotional-regulation techniques. The 10-Item Perceived Stress Scale (PSS-10) and the Active Coping subscale of the Brief Coping Orientation to Problems Experienced (COPE) inventory were administered before and after the intervention.

Results

Mean PSS-10 scores decreased significantly from 21.44 ± 3.58 to 15.84 ± 4.05 (mean reduction = 5.60 ± 1.87, 95% confidence interval (CI) 5.07-6.13; a 26.9% relative reduction; Wilcoxon signed-rank p < 0.001; Cohen’s d = 2.99). Active coping scores increased significantly from 4.22 ± 1.04 to 6.00 ± 1.28 (mean increase = 1.78 ± 0.76, 95% CI = 1.57-1.99; p < 0.001; Cohen’s d = 2.33). Every one of the 50 participants (100%) showed a reduction in PSS-10 score and a simultaneous increase in active coping. High-stress classifications fell from 10.0% to 0.0%, and low-stress classifications rose from 2.0% to 26.0%. The magnitude of stress reduction did not correlate significantly with the magnitude of increase in active coping (r = 0.108, p = 0.454).

Conclusions

A brief structured intervention delivered during clinical posting was associated with a large, uniform reduction in perceived stress and a corresponding improvement in active coping among MBBS students, supporting its incorporation into undergraduate clinical training programs.

## Introduction

Stress can be broadly understood as the physiological and psychological strain that results from demanding or threatening circumstances, appraised subjectively by the individual experiencing it [1]. Undergraduate clinical posts represent an important transition in medical education from a theoretical, classroom environment to interacting with real patients with time constraints and evaluation. This transition leads to an increase in stress due to unfamiliarity with clinical settings, differences between theory and practice, fear of mistakes, and the stress of dealing with patients [2]. Prolonged, uncontrolled stress can lead to anxiety, lack of confidence, emotional fatigue, and burnout, which adversely affect learning, academic performance, and future professional success [2,3].

There is considerable evidence that stress exists among nursing and medical students in different parts of the world, and the rates of moderate to significant stress are different in various settings [2-4]. In addition to describing levels of stress, some studies have focused on how students cope with stress by using active problem-solving strategies or passive emotion-focused strategies, which are usually measured by the Brief Coping Orientation to Problems Experienced (COPE) scale [2,5]. Research conducted in India and South Asia has shown that active coping strategies are the main methods of coping, as they are more effective than passive strategies; thus, it is better to develop these coping skills than to rely on the spontaneous development of these strategies by students [3,4].

Despite the existence of such research in the literature, studies that specifically teach students to cope with stress during clinical postings rather than merely examine the phenomenon of stress and the mechanisms of coping in hindsight are still scarce, especially in low-resource settings. Some studies conducted in universities during the COVID-19 pandemic showed that short training sessions on different methods of coping with stress through problem-solving techniques, emotional regulation, and social support resulted in a considerable decrease in perceived stress for students from various domains [6]. Second, some studies reported that a single-session workshop can increase students’ knowledge of coping, their confidence, and their application of adaptive coping strategies upon entering their clinical posts even when it is the only training they receive [7]. Systematic reviews and meta-analyses have highlighted mindfulness techniques and confirmed that structured psychological programs with varying formats can lead to a reduction in the level of stress for medical students [8-10].

This evidence indicates that a short structured program implemented into an existing clinical curriculum can contribute to a reduction in the level of stress among undergraduate medical students and not require any additional resource investment. This study aims to determine if such programs, which were delivered in a limited number of sessions during the undergraduate clinical posting in Medicine, can help reduce the level of stress and improve active coping skills of students. The primary objective was to assess the change in perceived stress levels, measured using the 10-Item Perceived Stress Scale (PSS-10), before and after the intervention. The secondary objective was to examine the accompanying change in active coping, assessed using the Active Coping subscale of the Brief COPE inventory.

## Materials and methods

Study design and setting

This was a pre-post interventional study conducted over a period of six to eight weeks in the Department of General Medicine in a tertiary care center in Tamil Nadu, India.

Study population and sampling

The study population comprised undergraduate MBBS students (third year and final year) undergoing their clinical posting in Medicine during the study period. Students who provided informed consent and were available for the full duration of the intervention were included. Students who did not consent, or who were absent for more than 50% of the intervention sessions, were excluded. A total of 50 students who completed both the pre- and post-intervention assessments were included in the final analysis, consistent with the feasible sample size of 40-60 students estimated a priori for detecting a moderate effect size in a pre-post design.

Instruments

Perceived stress was measured using PSS-10, originally derived from Cohen et al.’s 14-item scale and among the most widely used self-report instruments for assessing the degree to which situations in one’s life are appraised as unpredictable, uncontrollable, and overloading [1]. Respondents rate the frequency of stress-related thoughts and feelings over the preceding month on a five-point scale (0 = never to 4 = very often), yielding a total score ranging from 0 to 40. Consistent with established scoring conventions, total scores were categorized as low (0-13), moderate (14-26), or high (27-40) perceived stress [6]. Coping was assessed using the Active Coping subscale of the Brief COPE inventory, a widely validated abbreviated version of the original COPE inventory that captures the extent to which respondents take concrete, direct action to address the source of a stressor [5].

Intervention

Following the pre-intervention assessment, participants underwent a brief structured program delivered over two to four weeks, comprising three to four sessions of 30-45 minutes each: (i) an orientation session on understanding stress in the clinical setting; (ii) a coping skills training session covering problem-solving and time management techniques; (iii) a session on emotional-regulation techniques, including basic mindfulness and relaxation exercises; and (iv) an optional concluding session of guided reflection and debriefing. This format is consistent with brief workshop-style interventions previously reported to improve medical students’ coping knowledge and skills [7], and with the broader category of short-duration, low-resource psychological interventions evaluated in systematic reviews of medical student stress management [8-10]. The intervention was designed as a curriculum-embedded program feasible for delivery within routine clinical posting schedules, rather than as a separate extracurricular wellness activity.

Intervention schedule

The sessions were planned with flexibility in duration, accommodating the minimum and maximum time available in view of posting constraints. To minimize disruption to routine ward postings, each session was conducted for 45 minutes on weekends over a period of four consecutive weeks. The fourth session incorporated reflection and debriefing, thereby ensuring consolidation of learning. In total, four sessions were delivered, and the overall intervention period spanned one month.

Intervention team

The intervention was conducted by a multidisciplinary team with diverse expertise. The team comprised a Professor of General Medicine, who served as the Principal Investigator; an Associate Professor of Psychiatry; a Professor of Pharmacology; and an Assistant Professor of Community Medicine. All faculty members possessed MD qualifications, ensuring subject matter competence across clinical and academic domains. In addition, a psychiatric stress counsellor posted in the department was included in the team, providing specialized counselling support and enhancing the psychosocial dimension of the intervention.

Data collection procedure

The PSS-10 and Brief COPE Active Coping subscale were self-administered by participants immediately before the start of the intervention (pre-assessment) and again immediately after completion of all sessions (post-assessment). Responses were anonymized and coded using unique student identifiers to permit paired pre-post comparison while maintaining confidentiality.

Statistical analysis

The data were entered in Microsoft Excel (Microsoft Corp., Redmond, WA, USA) and subsequently analyzed using statistical software. Descriptive statistics such as mean, standard deviation (SD), median, interquartile range (IQR), and range were calculated for the scores for PSS-10 and Active Coping at two different time points. As the Shapiro-Wilk test showed that there was no normal distribution of data for the pre-post difference scores for PSS-10 (W = 0.916, p = 0.002) and Active Coping (W = 0.788, p < 0.001), the non-parametric Wilcoxon signed-rank test was utilized as the primary statistical test to perform paired comparisons of the data with additional reporting of a paired t-test and 95% confidence intervals (CIs) for the mean difference for completeness. Effect size was estimated using Cohen’s d for paired samples. The proportion of individual students showing an improvement, no change, or worsening on each measure was also calculated. The relationship between the magnitude of change in perceived stress and the magnitude of change in active coping was examined using Pearson correlation. A p-value <0.05 was considered statistically significant.

Ethical considerations

Ethical standards for the study were adhered to in accordance with the Declaration of Helsinki. Institutional ethical approval and permission were secured before the study began. Participation was entirely voluntary and written informed consent was obtained from all participants after they had been given a participant information sheet and adequate time to make a decision. Participants were made aware of their right to withdraw from the study at any time without any academic impact. Data were anonymized and coded, and access was limited to the investigators, with data stored in a secure manner. The intervention was deemed to present minimal risk; however, as a precaution, support or referral was available if any distress was observed during any of the sessions.

## Results

In total, students completed both the pre- and post-intervention assessments and were included in the analysis. All participants had complete paired data for both PSS-10 and Active Coping scores, giving a paired analytic sample of n = 50 with no missing values on either measure at either time point.

Perceived stress

The mean PSS-10 score decreased from 21.44 ± 3.58 (median = 21, IQR = 19.25-24; range = 12-30) before the intervention to 15.84 ± 4.05 (median = 15, IQR = 13.25-18; range = 5-26) after the intervention, representing a mean reduction of 5.60 ± 1.87 points (95% CI = 5.07-6.13), equivalent to a 26.9% ± 10.3% relative reduction from baseline (Table 1).

**Table 1:** Descriptive statistics of PSS-10 and active coping scores before and after the intervention (n = 50). PSS-10 score interpretation: Higher scores indicate greater perceived stress. Active coping score interpretation: Higher scores indicate better coping strategies. PSS-10: Perceived Stress Scale – 10-item version; Brief COPE: Brief Coping Orientation to Problems Experienced scale; IQR: interquartile range

Variable	Pre‑intervention, mean ± SD	Post‑intervention, mean ± SD	Pre-intervention, median (IQR)	Post-intervention, median (IQR)	Pre-intervention, range	Post-intervention, range	Mean difference ± SD
PSS‑10 score	21.44 ± 3.58	15.84 ± 4.05	21 (19.25–24)	15 (13.25–18)	12–30	5–26	5.60 ± 1.87
Active coping (Brief COPE)	4.22 ± 1.04	6.00 ± 1.28	4 (3–5)	6 (5–7)	3–6	4–8	1.78 ± 0.76

This reduction was statistically significant on both the paired t-test (t = 21.14, df = 49, p < 0.001) and the Wilcoxon signed-rank test (p < 0.001), with a very large effect size (Cohen’s d = 2.99) (Table 2).

**Table 2:** Inferential statistics for pre-post comparisons (n = 50). Paired t (df = 49) denotes the paired t-test statistic with 49 degrees of freedom used to compare pre- and post-intervention mean scores. df = n−1. Wilcoxon W represents the Wilcoxon signed-rank test statistic for non-parametric paired comparisons. P-values indicate the probability of observing the results under the null hypothesis. *: P-values <0.05 were considered statistically significant. PSS-10: Perceived Stress Scale – 10-item version; CI: confidence interval

Variable	Paired t (df = 49)	P-value	Wilcoxon W	P-value	95% CI of mean difference	Cohen’s d
PSS-10 (pre vs. post)	21.14	<0.001	0	<0.001	5.07–6.13	2.99
Active coping (pre vs. post)	16.48	<0.001	0	<0.001	1.57–1.99	2.33

At the individual level, all 50 (100%) participants showed a reduction in PSS-10 score following the intervention; no student showed no change or a worsening of perceived stress. The magnitude of individual reduction ranged from 2 to 12 points, with a relative reduction ranging from 11.1% to 58.3% of the baseline score.

When classified according to standard PSS-10 categories, the proportion of students with high perceived stress fell from 10.0% (5/50) before the intervention to 0.0% (0/50) after the intervention, while the proportion with low perceived stress rose from 2.0% (1/50) to 26.0% (13/50). The proportion classified as moderately stressed decreased from 88.0% (44/50) to 74.0% (37/50) (Table 3).

**Table 3:** Distribution of perceived stress categories before and after the intervention (n = 50). Perceived stress categories: Low (0–13), moderate (14–26), and high (27–40). Participants (n = 50) showed a shift from moderate/high stress to lower stress levels after the program. Highlights the effectiveness of the intervention in reducing perceived stress.

Perceived stress category	Pre-intervention, n (%)	Post-intervention, n (%)
Low (0–13)	1 (2.0%)	13 (26.0%)
Moderate (14–26)	44 (88.0%)	37 (74.0%)
High (27–40)	5 (10.0%)	0 (0.0%)
Total	50 (100.0%)	50 (100.0%)

The magnitude of PSS-10 reduction was broadly similar across baseline stress categories: the single student with low baseline stress showed a reduction of 7 points; the 44 students with moderate baseline stress showed a mean reduction of 5.55 ± 1.85 points; and the five students with high baseline stress showed a mean reduction of 5.80 ± 2.39 points (Table 4), indicating that the benefit of the intervention was not confined to students who began with the highest stress scores.

**Table 4:** Mean PSS-10 reduction stratified by baseline stress category. *: Single case in the low‑stress category (n = 1). Participants were grouped as low (0–13), moderate (14–26), and high (27–40) based on initial PSS‑10 scores. The mean reduction was greatest in the low‑stress category (single case), while the moderate- and high‑stress groups demonstrated comparable improvements (5.55 ± 1.85 and 5.80 ± 2.39, respectively), indicating a consistent beneficial effect of the intervention across stress levels. PSS-10: Perceived Stress Scale – 10-item version

Baseline stress category	n	Mean PSS-10 reduction ± SD
Low (0–13)	1	7.00
Moderate (14–26)	44	5.55 ± 1.85
High (27–40)	5	5.80 ± 2.39

Active coping

The mean Active Coping score increased from 4.22 ± 1.04 (median = 4, IQR = 3-5; range = 3-6) before the intervention to 6.00 ± 1.28 (median = 6, IQR = 5-7; range = 4-8) after the intervention, a mean increase of 1.78 ± 0.76 points (95% CI = 1.57-1.99) (Table 1). This increase was statistically significant on both the paired t-test (t = 16.48, df = 49, p < 0.001) and the Wilcoxon signed-rank test (p < 0.001), with a large effect size (Cohen’s d = 2.33) (Table 2). As with perceived stress, all 50 (100%) participants showed an increase in Active Coping score following the intervention, with no student showing no change or a decline.

Relationship between change in stress and change in coping

**Figure 1:**
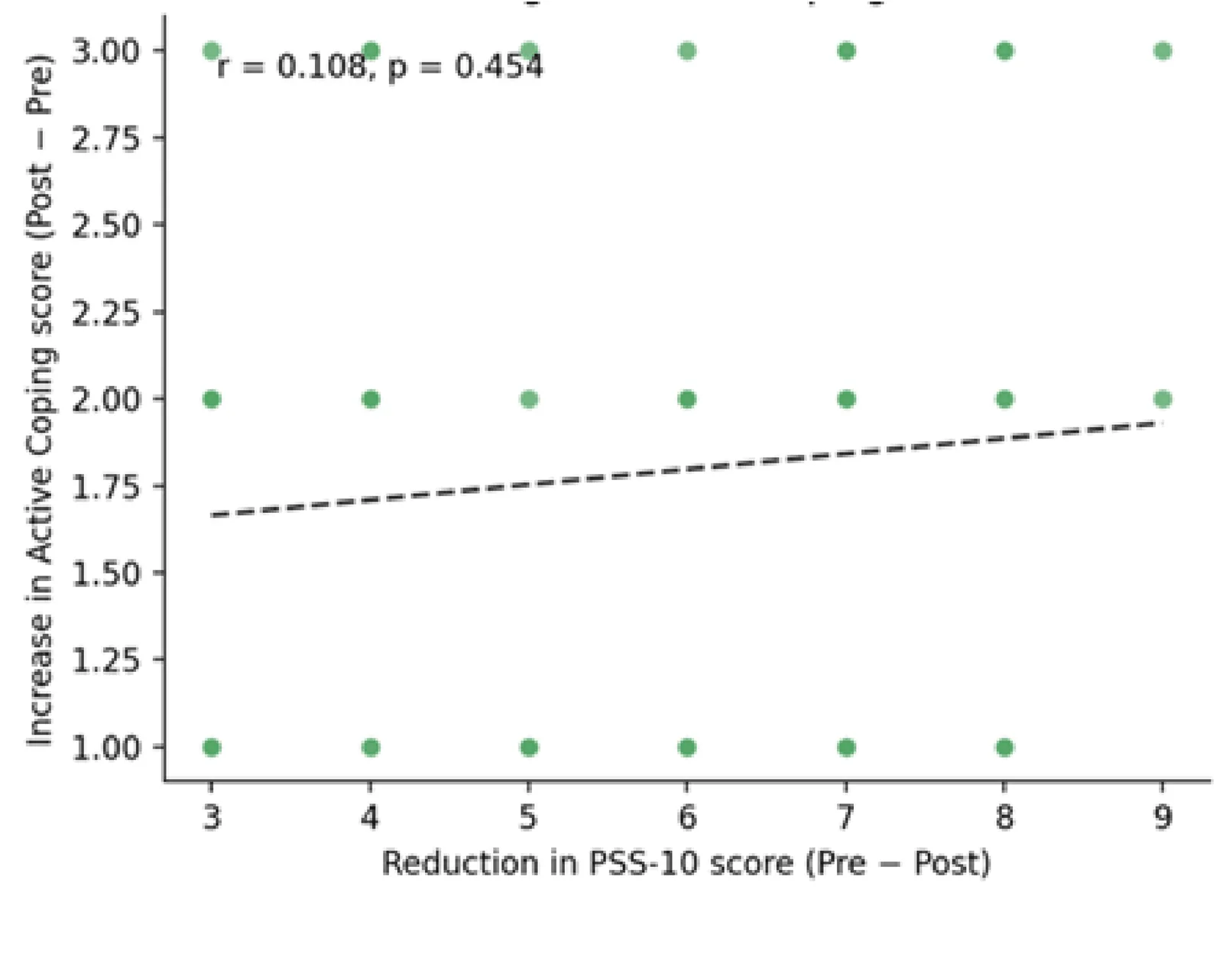
Relationship between change in perceived stress and change in active coping (n = 50). Scatter plot of the relationship between the magnitude of reduction in PSS-10 score and the magnitude of increase in Active Coping score (n = 50). PSS-10: Perceived Stress Scale – 10-item version

The magnitude of reduction in PSS-10 score was not significantly correlated with the magnitude of increase in Active Coping score (Pearson r = 0.108, p = 0.454) (Figure 1), suggesting that the improvement in active coping behaviors and the reduction in perceived stress, while both significant and near-universal at the individual level, did not track proportionally with one another: students with the largest gains in active coping were not necessarily those with the largest reductions in perceived stress.

## Discussion

The results of this interventional study indicate that the use of a brief structured program, embedded within the clinical posting period of the undergraduate Medicine course, has substantially decreased perceived stress and enhanced active coping skills among the participating MBBS students. Indeed, the average correlation coefficient found across participants was 0.952, while the mean difference of more than five points recorded on the PSS-10 scale was phenomenal, given the short duration of the intervention (two to four weeks) and its budgeting within a clinical training period schedule.

Results of this study were consistent with those of a similar study conducted during the COVID-19 pandemic among Egyptian university students, which, after the application of the structured program, noted a significant decline in the perceived stress levels of the student population, with positive responses recorded across all categories of student subgroups [6]. The consistency of this direction of effect across different student populations, stressor contexts, and cultural settings lends support to the premise that structured, skills-based coping interventions can produce meaningful reductions in perceived stress irrespective of the specific source of stress, irrespective of pandemic-related disruption or the demands of clinical training. Similarly, a brief single-session workshop-based intervention for medical students entering clerkships elsewhere has been shown to improve students’ knowledge, confidence, and self-reported use of adaptive coping strategies at both immediate and three-month follow-up, suggesting that even shorter interventions than the one used in the present study can shift students’ coping repertoire, though such studies have generally measured knowledge and attitudes rather than validated stress scores directly [7].

The parallel increase in active coping scores observed in this study aligns with descriptive findings from studies of nursing and medical students, in which active, problem-solving coping approaches were consistently among the most commonly employed strategies and were positively associated with favorable stress-related outcomes, in contrast to passive or avoidant strategies which showed no such association [3,4]. A South Indian cross-sectional study of first-year medical undergraduates similarly found active coping to be a prominent strategy among students under stress, alongside other emotion-focused approaches such as humor and religious coping [4]. Taken together with the present findings, this supports the rationale for interventions that directly cultivate problem-solving and active coping skills, rather than relying on students to develop these skills spontaneously through unstructured clinical exposure.

Interestingly, the magnitude of stress reduction in the present study did not correlate significantly with the magnitude of improvement in active coping at the individual level, despite both outcomes improving in every participant. This suggests that although active coping and perceived stress both moved in the favorable direction for the cohort as a whole, the pathway by which the intervention reduced stress for any given student may not have been mediated solely, or at least not proportionally, through active coping as measured by this single Brief COPE subscale. Other components of the intervention, such as the emotional regulation and reflective debrief sessions, or unmeasured factors such as peer support generated through group sessions, may have contributed independently to stress reduction. This is broadly consistent with the multifactorial sources of clinical stress described among nursing and medical students, spanning direct patient care, interactions with teaching staff, workload, peer comparison, and unfamiliarity with the clinical environment, which suggests that a single coping domain is unlikely to fully mediate stress outcomes [2,3].

The broader literature on structured psychological interventions for medical student stress, most extensively developed around mindfulness-based approaches, offers a useful point of comparison for the magnitude of effect observed here. Systematic reviews and meta-analyses of mindfulness-based interventions in medical students have generally reported small-to-moderate standardized effect sizes on stress outcomes, in the range of d = 0.29 to d = 0.37 for controlled and one-arm pre-post designs, respectively [9,10]. The considerably larger effect size observed in the present single-arm study (d = 2.99 for PSS-10) should therefore be interpreted with caution: it may reflect a genuinely stronger effect of the multicomponent intervention used here, but is equally likely to be inflated by the absence of a control group, the small single-center sample, practice effects on repeated administration of the PSS-10 over a short interval, and the general tendency of single-arm, pre-post designs to overestimate true intervention effects relative to randomized, controlled designs [8-10]. A comparable pattern of universal, large-magnitude improvement has been reported in single-institution, single-arm, pre-post workshop evaluations among medical students, which similarly lack a comparison group [7].

Comparison with the broader Indian literature on medical student stress underscores the scale of the problem this intervention sought to address. Cross-sectional studies from Indian medical colleges have reported moderate-to-high perceived stress in the majority of undergraduates, with estimates for the proportion experiencing at least moderate stress ranging from around two-thirds to over 90% depending on the institution, year of study, and instrument used [11,12]. There have been reports of similar stress, anxiety, and depression in the Egyptian population and other South Asian medical student groups, as shown by the use of PSS and other such instruments in those populations [13,14]. With the reduction in the level of stress among the subjects after intervention, it shows the ability of such interventions to bring a notable change in the results caused by other means of coping with stress.

Implications for curriculum design include the introduction of low-resource modules for coping with stress alongside control clinical postings, which now represents an essential part of medical students’ training as opposed to creating a separate wellness program. Systematic reviews on medical students’ stress management programs have also supported the inclusion of resilience training, mindfulness techniques, and other wellness workshops as important factors contributing to the success of resilience-building programs for medical students [15].

Limitations

This study has several limitations. The single-arm, pre-post design without a concurrent control group precludes definitive attribution of the observed changes to the intervention itself, as opposed to natural adaptation to the clinical environment over time, regression to the mean, or the effect of repeated testing on the same instrument over a short interval. The finding that all 50 participants improved on both outcome measures, while encouraging, is also unusual enough to raise the possibility of response bias, social desirability in reporting to investigators who delivered the intervention, or a testing effect specific to this cohort, and should temper how far the observed effect sizes are generalized. The sample was drawn from a single department at a single institution, which may limit generalizability to other clinical settings and curricula. Reliance on self-reported measures introduces the possibility of social desirability bias, and the short post-intervention assessment window does not permit conclusions regarding the durability of the observed improvements. Coping was assessed using a single Brief COPE subscale (active coping) rather than the full inventory, limiting insight into how other coping domains, such as emotional support-seeking or avoidance, may have changed. Future research employing a randomized controlled design with an untreated comparison group, longer follow-up, and the complete Brief COPE inventory would help to confirm and extend these findings.

## Conclusions

A brief, structured, low-resource intervention delivered during undergraduate Medicine clinical posting was associated with a significant, large-magnitude, and near-universal reduction in perceived stress and an increase in active coping among MBBS students. These findings support the feasibility and potential value of embedding brief coping skills interventions within routine clinical training, though confirmation through controlled trials with longer follow-up is warranted before widespread implementation.

## References

[1] Cohen S, Kamarck T, Mermelstein R (1983). A global measure of perceived stress. J Health Soc Behav.

[2] Abaribe C, Uduhirinwa C, Onuiri A (2025). Perceived stress and coping mechanisms among nursing students during clinical placements in Babcock university, Ilishan-Remo, Ogun state. BMC Nurs.

[3] Paudel U, Parajuli A, Shrestha R, Kumari S, Adhikari Yadav S, Marahatta K (2022). Perceived stress, sources of stress and coping strategies among undergraduate medical students of Nepal: a cross-sectional study. F1000Res.

[4] Shakthivel N, Amarnath VM, Ahamed F, Rath RS, Sethuraman AR, Rizwan AS (2017). Level of perceived stress and coping strategies prevailing among 1st year medical undergraduate students: a cross-sectional study from South India. Int J Med Public Health.

[5] Carver CS (1997). You want to measure coping but your protocol's too long: consider the brief COPE. Int J Behav Med.

[6] Elsary AY, El-Sherbiny NA (2023). The impact of stress-coping strategies on perceived stress during the COVID-19 pandemic among university students an interventional study. BMC Psychiatry.

[7] Manning-Geist B, Meyer F, Chen J, Pelletier A, Kosman K, Chen XP, Johnson NR (2020). Pre-clinical stress management workshops increase medical students' knowledge and self-awareness of coping with stress. Med Sci Educ.

[8] Daya Z, Hearn JH (2018). Mindfulness interventions in medical education: a systematic review of their impact on medical student stress, depression, fatigue and burnout. Med Teach.

[9] Hathaisaard C, Wannarit K, Pattanaseri K (2022). Mindfulness-based interventions reducing and preventing stress and burnout in medical students: a systematic review and meta-analysis. Asian J Psychiatr.

[10] Sperling EL, Hulett JM, Sherwin LB, Thompson S, Bettencourt BA (2023). The effect of mindfulness interventions on stress in medical students: a systematic review and meta-analysis. PLoS One.

[11] Waghachavare VB, Dhumale GB, Kadam YR, Gore AD (2013). A study of stress among students of professional colleges from an urban area in India. Sultan Qaboos Univ Med J.

[12] Raja S, Balasubramanian G, Jamuna Rani R (2022). Prevalence of depression, anxiety and stress among private medical college students in South India: a cross-sectional study. J Educ Health Promot.

[13] Ebrahim OS, Sayed HA, Rabei S, Hegazy N (2024). Perceived stress and anxiety among medical students at Helwan University: a cross-sectional study. J Public Health Res.

[14] Melaku L, Bulcha G, Worku D (2021). Stress, anxiety, and depression among medical undergraduate students and their coping strategies. Educ Res Int.

[15] Bin Abdulrahman KA, Hefny M, Alghamdi SA (2025). The value of stress management programs for medical students: a systematic review. Front Public Health.

